# Association of accelerometer-measured physical activity, back static muscular endurance and abdominal obesity with radicular pain and non-specific low back pain

**DOI:** 10.1038/s41598-023-34733-4

**Published:** 2023-05-12

**Authors:** Munkh-Erdene Bayartai, Juhani Määttä, Jaro Karppinen, Petteri Oura, Jani Takatalo, Juha Auvinen, Korpelainen Raija, Maisa Niemelä, Hannu Luomajoki

**Affiliations:** 1 Institute of Physiotherapy, School of Health Professions, Zurich University of Applied Sciences, ZHAW, Katharina-Sulzer-Platz 9, 8401 Winterthur, Switzerland; 2 Medical Research Center Oulu, Oulu University Hospital and University of Oulu, Oulu, Finland; 3 Research Unit of Population Health, Faculty of Medicine, University of Oulu, Oulu, Finland; 4 Department of Physical Therapy, School of Nursing, Mongolian National University of Medical Sciences, Ulaanbaatar, Mongolia; 5 Department of Sports and Exercise Medicine, Oulu Deaconess Institute Foundation Sr., Oulu, Finland; 6 Research Unit of Medical Imaging, Physics and Technology, University of Oulu, Oulu, Finland; 7 Research Unit of Health Sciences and Technology, University of Oulu, Oulu, Finland

**Keywords:** Diseases, Health care, Medical research, Rheumatology

## Abstract

Low back pain (LBP) is the leading cause of disability worldwide and often associated with lifestyle factors. However, studies further examining the role of these lifestyle factors in non-specific low back pain in comparison with radicular pain are sparse. The aim of this cross sectional study was to investigate how diverse lifestyle factors are associated with LBP. The study population of 3385 middle aged adults with and without low back pain was drawn from a large Birth 1966 Cohort. Outcome measures were steps per day, abdominal obesity, physical activity and endurance of the back muscles. Back static muscular endurance, abdominal obesity and physical activity were measured by means of the Biering–Sørensen test, waist circumference and a wrist worn accelerometer, respectively. Logistic regression analysis was applied to estimate associations of back static muscular endurance, abdominal obesity and accelerometer-measured physical activity with non-specific low back pain and radicular pain. An additional 1000 steps per day were associated with 4% lower odds of having non-specific low back pain. Participants with abdominal obesity had 46% higher odds of having radicular pain, whereas increases of 10 s in back static muscular endurance and 10 min in daily vigorous physical activity were associated with 5% and 7% lower odds of having radicular pain, respectively. In this population-based study, non-specific low back pain and radicular pain were associated with different lifestyle and physical factors at midlife. Non-specific low back pain was associated only with the average daily number of steps, whereas abdominal obesity was the strongest determinant of radicular pain, followed by vigorous physical activity and back static muscular endurance. The findings of this study contribute to better understand the role of lifestyle factors in both non-specific low back pain and radicular pain. Future longitudinal studies are required to explore causality.

## Introduction

Low back pain is the leading cause of disability worldwide and one of the most common reasons people seeking health and medical services^1,2^. The global years lived with disability for low back pain increased by 52.7% from 42.5 million to 64.9 million between 1990 and 2017^3^. However, in most cases the exact cause of low back pain remains unclear^4^. Low back pain is multifactorial, and genetic, biophysical, psychological and social factors are considered to substantially contribute to it^5^. Additionally, lifestyle factors such as physical inactivity, obesity and smoking are also associated with the incidence of low back pain^5^. These factors also appear to play an important role in low back pain attributable to a recognizable, known specific pathology such as radicular pain^6^. Radicular pain, although not as prevalent as non-specific low back pain, is associated with poorer health outcomes in people with low back pain^7^. However, studies further examining the role of these lifestyle factors in non-specific low back pain in comparison with radicular pain are lacking.

Participation in physical activity (PA) and muscle-strengthening activities has well-established health benefits to both healthy people and individuals with obesity or various health conditions, including musculoskeletal disorders^8–12^. Reducing sedentary behavior and promoting PA, particularly in moderate levels of PA are generally believed to be beneficial for people with low back pain^13,14^. However, systematic reviews reported that most studies to date examining this relationship between PA and low back pain have used self-reported measures of PA^13,15^, although less precise than objective measurements to estimate PA^16^. Additionally, the effect of some types and intensities of PA, particularly high levels of PA on low back pain remain inconclusive due to inconsistent findings^13,17^. A 1-year prospective cohort study of 387 office workers showed that an increase in daily walking steps was associated with reduced risk of neck pain but not low back pain^18^, whereas a cross sectional study on 30 adults with and without low back pain reported that those with low back pain took fewer step and spent less time engaged in walking compared to healthy participants^19^. These results suggest the need for further studies using objective measurements of PA to explore its relationship with low back pain.

Low back pain also appears to be associated with muscle weakness. For instance, a cross sectional study of 101 female patients aged 60 and over reported that patients with low back pain had lower abdominal trunk muscle strength than those without low back pain^20^. A meta-analysis of six randomized controlled trials comparing the effect of progressive aerobic training to progressive resistance training on chronic non-specific low back pain showed that both training improved pain in individuals with chronic non-specific low back pain but neither aerobic training nor resistance training were superior to each other^21^. In terms of assessing muscle strength and endurance, the Biering–Sørensen test is found to be reliable and accurate^22^. However, studies investigating whether back muscle strength is differently associated with low back pain between its different types such as non-specific low back pain and radicular pain are lacking.

Most studies included in previous systematic reviews examining the relationship between obesity and low back pain have investigated general obesity rather than abdominal obesity in relation to low back pain^6,23^. There has been little research examining whether the association of low back pain with obesity differs between general and abdominal obesity. Abdominal obesity appears to play a more important role than general obesity in the risk of developing multiple health conditions, including cardiovascular disease, major adverse cardiac events and metabolic syndrome^24–26^. In addition, abdominal obesity is found to accelerate muscle strength decline and substantially increase spinal loads^27,28^. However, research on the association between abdominal obesity and low back pain is scarce.

### Purpose

The aim of this study was to explore the association of back static muscular endurance, accelerometer measured PA and abdominal obesity with radicular pain and non-specific low back pain in a large-population-based setting. The main hypothesis was that radicular pain and non-specific low back pain are associated with different lifestyle and physical factors.

## Methods

### Study population

**Figure 1:**
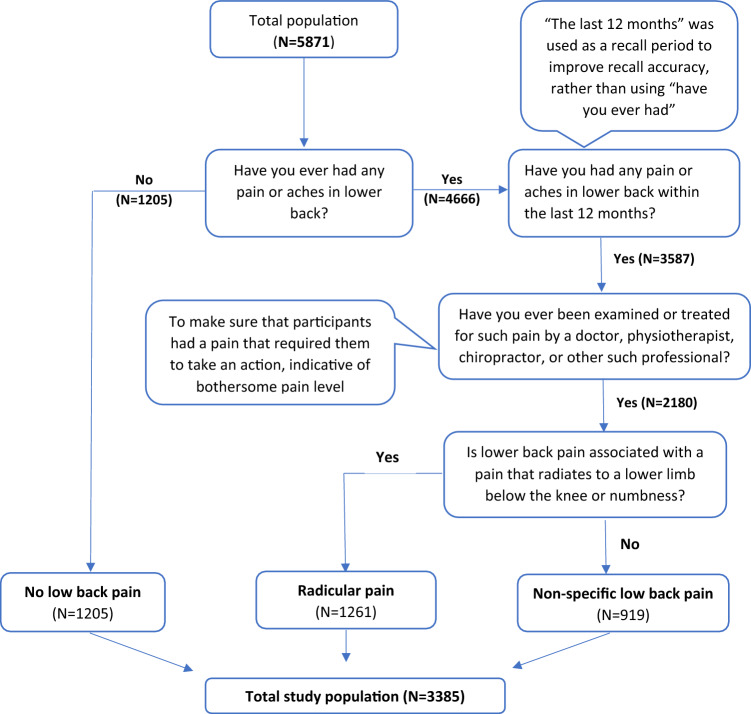
Flow chart of the study population.

A total of 5871 middle aged adults with and without low back pain were drawn from Birth Cohort people born 1966, and 3385 of them were eligible for the current study. The cohort initially comprised 12,231 children born in 1966^29^. The study was approved by the local Ethical Committee and was performed in accordance with the Declaration of Helsinki. The participants and their parents provided written informed consent for the study. Follow ups of this cohort occurred at 1, 14, 31 and 46 years of age, and data from the latest follow up conducted in the years between 2012 and 2014 were analyzed in the present cross-sectional study. Information related to this cohort and the follow-ups is described in detail elsewhere^30^. Figure 1 is a flow chart of the present study.

### Assessment of low back pain

Participants were initially assigned into two groups, namely “No low back pain” and “Low back pain” by asking whether they had any pain or aches in lower back. Those who answered “Yes” and visited a doctor or other health professional for the examination or treatment of their low back pain within the past 12 months were then further categorized into two low back pain groups “Radicular pain” and “Non-specific low back pain” based on the questions related to back pain, as shown in Fig. 1.

### General and abdominal obesity

Nurses trained for the examination measured the anthropometric parameters of the cohort members such as body weight, height and waist circumference^30^. Body mass index (kg/m^2^) was calculated as body mass divided by height squared. General obesity was determined as a body mass index of ≥ 30 kg/m^2^, whilst abdominal obesity (central obesity) was defined as waist circumferences of ≥ 80 cm in women and ≥ 94 cm in men^31^.

### Back static muscular endurance

Back static muscular endurance was measured by the trained nurses using the Biering–Sørensen test, previously defined as an accurate method for the measurement of trunk muscles and endurance^22^. In the test, the participant is placed prone with the iliac crests positioned at the edge of the table, the buttocks as well as legs fixed to it and asked to hold the unsupported upper part of the body in a horizontal position^32^. Participants who had acute low back pain preventing them from performing the test were excluded. The test ends if the participant could no longer maintain the horizontal posture or reached his/her limit of tolerance for fatigue related symptoms. The hold time is recorded with a maximum test time of 240 s, considered the maximum duration that participants could maintain the horizontal posture^33^.

### Physical activity

A waterproof wrist-worn accelerometer Polar Active was used to measure PA. Polar Active has been shown to be accurate for measuring energy expenditure, compared against indirect calorimetry^34^ and doubly labeled water technique^35^. Participants were instructed to wear the Polar Active on the non-dominant hand 24 h per day, for a period of 14 days^36^. Measured PA was then categorized based on metabolic equivalent (MET) values into five different intensities of PA defined by device manufacturer, namely very light/sedentary behavior (1–2 MET), light (2–3.5 MET), moderate (3.5–5 MET), vigorous (5–8 MET), and very vigorous (≥ 8 MET). In earlier comparison study, these intensity levels for Polar Active provided more comparable results than often used limits defined for hip-worn accelerometers (sedentary behavior ≤ 1.5 METs, light PA 1.5–3 METs, moderate PA as 3–6 METs) for sedentary time and light and moderate intensity activity levels when using the above-mentioned limits for ActiGraph (model GT3X)^37^. The average daily duration spent on each intensity level was calculated and expressed as minutes per day. The average number of steps taken per day was also determined in addition to the average duration of these different intensities of PA. Very light (sedentary behavior), light, moderate, vigorous, very vigorous and moderate to vigorous (≥ 3.5 METs, MVPA) intensities of PA and the average number of steps were analyzed in the current study. Participants providing at least 4 valid days defined as accelerometer wear time during waking hours at least 10 h were included in the analyses.

### Confounding factors

Sex, general obesity, smoking status and physical strenuousness of work were considered as confounding factors^5,38^. Responses to the question “have you ever smoked?” were used to categorize participants into two groups, namely never smokers and smokers (including current and ex-smoker). The question “To what extent are the following tasks and postures part of your job?”, comprised of 9 items associated with work, was used to determine participants’ physical strenuousness of work. These items include (1) Heavy physical work in which the body has to struggle. (2) Lifting loads of 1–15 kg. (3) Lifting loads over 15 kg. (4) Continuous movement or walking. (5) Repetitious work movements. (6) Standing. (7) Working with the upper arms elevated. (8) Forward-bent work postures and (9) rotational movements of the trunk. Participants were asked to score each item on a Likert-like scale of 1 (not at all or very rarely) to 5 (very often). The scale was reclassified as physically strenuous work (3–5 points) or light work (1–2 points). The medians were then used as cutoffs to classify the sum of nine items into physically light work and strenuous work^38^.

### Statistical analysis

Descriptive statistics and inferential analyses were performed using R version 3.6.0^22^. In the descriptive statistics, mean values and standard deviations (SD) for the participant characteristics of body mass index, waist circumference, back static muscular endurance and PA were determined. The Shapiro–Wilk test was applied to check for data normality. Analysis of variance (ANOVA) for normally distributed data and Kruskal–Wallis for non-normally distributed parameters were employed to compare differences between groups. The Chi-square test was used for categorical variables. Multivariable logistic regression analysis was applied to determine associations of back static muscular endurance, obesity and PA with radicular pain and non-specific low back pain. To account for confounding factors, logistic regression models analyzing the association between waist circumference, abdominal obesity and low back pain were adjusted for sex, smoking status and physical strenuousness of work, whilst the association of PA, as well as back static muscular endurance with low back pain was adjusted for general obesity in addition to sex, smoking status and physical strenuousness of work. PA variables were included separately in the multivariable logistic regression models to avoid multicollinearity. Observations with missing data were excluded from the analysis. *p* values less than 0.05 were considered to be statistically significant.

### Ethics approval and consent to participate

The study was approved by the Ethical Committee of the Northern Ostrobothnia Hospital District in Oulu, Finland (94/2011) and was performed in accordance with the Declaration of Helsinki. The participants and their parents provided written consent for the study.

## Results

Data of 3385 individuals with and without low back pain were analyzed in the current study (Table 1).

**Table 1:** The characteristics (mean [SD]) of the middle aged study participants (N = 3385).

No	Variables	No low back pain (N = 1205)	Radicular pain (N = 1261)	Non-specific low back pain (N = 919)	p value
1	Sex (female)	56%	54%	52%	0.23^c^
2	Smoking status (smokers)	53.6%	64.1%	56.1%	< 0.001^c^
3	Body mass index (kg/m^2^)	26.7 (4.9)	27.3 (4.8)	26.8 (5.0)	< 0.001^k^
4	Waist circumference (cm)	90.8 (13.9)	93.2 (13.3)	91.6 (13.5)	< 0.001^k^
5	Back static muscular endurance (sec)	179.9 (61.3)	160.2 (65.1)	175.7 (58.2)	< 0.001^k^
6	Physical strenuousness of work (strenuous)	47%	56%	52%	< 0.001^c^
7	Duration of pain (more than 30 days) within the last 12 months	–	38%	35%	0.19^c^
8	PA				
	Very light PA/sedentary behavior (min per day)	630.5 (93.8)	621.8 (94.3)	637.8 (90.8)	0.001^k^
	Light PA (min per day)	277.3 (72.4)	287.6 (75.8)	271.4 (71.5)	< 0.001^k^
	Moderate PA (min per day)	36.3 (23.2)	37.5 (23.8)	37.4 (22.1)	0.33^k^
	Vigorous PA (min per day)	24.7 (16.1)	22.4 (15.9)	22.9 (15.6)	< 0.001^k^
	Very vigorous PA (min per day)	8.5 (10.8)	7.4 (9.0)	8.6 (11.2)	0.008^k^
	MVPA (min per day)	69.5 (34.7)	67.3 (34.7)	68.9 (34.7)	0.27^k^
	Steps (N per day)	10,959.2 (3621.3)	10,744.7 (3851.6)	10,378.3 (3679.3)	0.006^k^

p value-statistical significance computed by using Kruskal–Wallis test^k^ and Chi-square test^c^ for comparison between the three groups.

*SD* standard deviation, *PA* physical activity, *MVPA* moderate to vigorous physical activity.

The number of smokers was greater among participants with radicular pain and non-specific low back pain than those without low back pain. Compared with participants without low back pain, those with radicular pain and non-specific low back pain also had higher body mass index and waist circumference but lower back muscle endurance and the average daily number of steps as well as less participation in vigorous PA (Table 1).

### Association of abdominal obesity, back static muscular endurance and PA with radicular pain and non-specific low back pain

The average daily number of steps was the only factor associated with non-specific low back pain (Table 2). An additional 1000 steps per day were associated with 4% lower odds of having non-specific low back pain. Among the explanatory variables associated with radicular pain, abdominal obesity was the strongest determinant. Participants with abdominal obesity had 46% higher odds of having radicular pain, whereas increases of 10 s in back static muscular endurance and 10 min in daily vigorous PA were associated with 5% and 7% lower odds of having radicular pain, respectively.

**Table 2:** Association of waist circumference, back static muscular endurance and PA with low back pain, estimated with multivariable logistic regression among middle aged participants (N = 3385).

No.	Variables	OR	95% CI	OR^a^	95% CI	OR^b^	95% CI	OR^c^	95% CI
Non-specific low back pain (NSLBP vs. NoLBP)									
1	Waist circumference (10 cm)	1.04	0.97–1.12	1.00	0.93–1.08	–	–	0.98	0.88–1.09
2	Abdominal obesity	1.09	0.89–1.33	1.06	0.86–1.69	–	–	1.04	0.84–1.30
3	Back static muscular endurance (10 s)	0.98	0.97–1.00	0.99	0.97–1.01	0.99	0.97–1.01	0.99	0.97–1.01
4	PA								
	Very light PA/sedentary behavior (10 min per day)	1.01	0.99–1.02	1.01	0.99–1.02	1.01	0.99–1.02	1.01	0.99–1.02
	Light PA (10 min per day)	0.99	0.97–1.00	0.99	0.98–1.01	0.99	0.98–1.01	0.98	0.97–1.00
	Moderate PA (10 min per day)	1.02	0.98–1.07	1.00	0.95–1.05	1.00	0.95–1.05	0.99	0.94–1.05
	Vigorous PA (10 min per day)	**0.93**	0.87–0.99*	0.95	0.89–1.01	0.95	0.89–1.01	0.95	0.88–1.01
	Very vigorous PA (10 min per day)	1.01	0.92–1.10	0.97	0.88–1.07	0.98	0.88–1.07	0.97	0.88–1.06
	MVPA (10 min per day)	0.99	0.96–1.02	0.98	0.95–1.01	0.98	0.96–1.01	0.98	0.95–1.02
	Steps (1000 steps per day)	**0.95**	0.93–0.98*	**0.96**	0.93–0.99*	**0.96**	0.93–0.99*	**0.97**	0.93–0.98*
5	Confounding factors								
	General obesity (BMI ≥ 30 kg/m^2^)	1.05	0.83–1.33	–	–	–	–	–	–
	Sex (female)	0.86	0.72–1.02	–	–	–	–	–	–
	Smoking status (smokers)	1.10	0.92–1.30	–	–	–	–	–	–
	Physical strenuousness of work	**1.22**	1.02–1.44*	–	–	–	–	–	–
Radicular pain (RBP vs. NoLBP)									
1	Waist circumference (10 cm)	**1.14**	1.07–1.22**	**1.12**	1.05–1.21**	–	–	**1.13**	1.02–1.25*
2	Abdominal obesity	**1.53**	1.26–1.87**	**1.46**	1.19–1.78**	–	–	**1.40**	1.13–1.73*
3	Back strength (10 s)	**0.95**	0.93–0.96**	**0.95**	0.94–0.97**	**0.95**	0.94–0.97**		
4	PA								
	Very light PA/sedentary behavior (10 min per day)	**0.99**	0.98–0.99*	**0.99**	0.98–0.99*	**0.99**	0.98–0.99*	0.99	0.98–1.00
	Light PA (10 min per day)	**1.02**	1.01–1.03*	**1.02**	1.01–1.03*	**1.02**	1.01–1.03**	**1.01**	1.00–1.03*
	Moderate PA (10 min per day)	1.02	0.98–1.06	1.01	0.97–1.06	1.02	0.98–1.07	1.01	0.96–1.05
	Vigorous PA (10 min per day)	**0.91**	0.86–0.97*	**0.92**	0.87–0.98*	**0.93**	0.88–0.99*	**0.93**	0.87–0.98*
	Very vigorous PA (10 min per day)	**0.89**	0.81–0.98*	**0.87**	0.78–0.97*	**0.89**	0.80–0.98*	**0.88**	0.79–0.97*
	MVPA (10 min per day)	0.98	0.95–1.01	0.99	0.97–1.01	0.98	0.96–1.01	0.98	0.95–1.01
	Steps (1000 steps per day)	0.98	0.96–1.01	0.99	0.96–1.01	0.99	0.97–1.02	0.99	0.96–1.01
5	Confounding factors								
	General obesity (BMI ≥ 30 kg/m^2^)	**1.29**	1.04–1.61*	–	–	–	–		
	Sex (female)	0.93	0.79–1.09	–	–	–	–		
	Smoking status (smokers)	**1.54**	1.31–1.81**	–	–	–	–		
	Physical strenuousness of work	**1.44**	1.23–1.69**	–	–	–	–		

*OR* odds ratio, *CI* confidence interval, *PA* physical activity, *MVPA* moderate to vigorous physical activity, *NSLBP* non-specific low back pain, *NoLBP* no low back pain, *RBP* radicular pain.

Significant values are in bold.

^a^Adjusted for sex and smoking status.

^b^Adjusted for general obesity status in addition to ^a^.

^c^Adjusted for physical strenuousness of work in addition to ^b^.

*p < 0.05, **p < 0.001.

## Discussion

The purpose of the present study was to examine the association of back static muscular endurance, abdominal obesity and accelerometer-measured PA with radicular pain and non-specific low back pain in a large population-based cohort. The main finding was that non-specific low back pain and radicular pain were associated with different lifestyle and physical factors. Non-specific low back pain was associated only with the average daily number of steps, whereas abdominal obesity was the strongest determinant of radicular pain, followed by vigorous PA and back muscle strength measured with static endurance test.

The average daily number of steps was associated with non-specific low back pain. A previous systematic review examining the relationship between PA and low back pain reported that undertaking moderate levels of PA is beneficial to people with low back pain^13^. However, another systematic review of cross sectional and prospective cohort studies examining different types and intensities of PA in relation to low back pain obtained conflicting findings for the association between PA and low back pain^17^, implying inconsistency in the association of PA with low back pain. These inconsistent findings could be due to heterogeneity between studies with different sample sizes, the complex and multidimensional nature of PA and the lack of usage of rigorous and accurate methods for assessment of PA, such as device-based measurements of PA. The current study had a large sample size to explore this association using accelerometer measured PA, with the key finding that the average daily number of steps was associated with non-specific low back pain, whereas no statistically significant associations were found between different intensities of PA with non-specific low back pain. A previous observational study of 30 participants comparing a difference in accelerometer-measured PA between individuals with and without low back pain reported that the chronic back pain group took 3480 fewer daily steps than those without low back pain^19^. This previous study, although having a small sample size, was in line with the findings from the present study. These results suggest that a total daily step count rather than intensity of activity could be a superior determinant of non-specific low back pain.

Abdominal obesity was the strongest determinant of radicular pain among the lifestyle factors assessed in current study. This result was anticipated, as a previous meta-analysis of 26 cross-sectional, case–control and cohort studies reported the positive relationship between obesity and sciatica^6^. Most studies included in this meta-analysis predominantly investigated general obesity in relation to radicular pain as opposed to abdominal obesity. The present study found that participants with abdominal obesity had 46% higher odds of having radicular pain, whilst general obesity was associated with 29% higher odds of radicular pain. However, none of these obesity measurements were associated with non-specific low back pain. This was in agreement with a previous study conducted in young adults that showed general obesity and abdominal obesity to be associated with radicular pain but not with non-specific low back pain^39^. These results suggest that abdominal obesity could be a better determinant of radicular pain than general obesity.

The present study also found that light, vigorous and very vigorous PA and back static muscular endurance were associated with radicular pain. To date, only a few studies have specifically investigated the association between PA and radicular pain relative to non-specific low back pain. Light PA was positively associated with radicular pain but the magnitude of odds ratio was small. The effect of PA on low back pain appears to differ depending on the intensity and domain of PA^40^. Therefore, the positive association between light intensity PA and radicular pain could be explained by PA domains linked with low back pain. For example, a previous twin study showed that heavy domestic PA was associated with an increased odds of low back pain and the magnitude of odds increased with the combination of heavy domestic and recreational PA^41^. However, the present study did not investigate different domains of PA, suggesting the importance of accounting for different domains of PA to better understand the association between light PA and radicular pain in future studies.

Regarding vigorous and very vigorous PA, a meta-analysis of six prospective studies demonstrated an inverse association between high level of leisure-time PA and lumbar radicular pain^42^, supporting the association of vigorous and very vigorous PA with radicular pain observed in the present study. However, the cross-sectional studies included in this systematic review showed conflicting findings for the association between PA and radicular pain, suggesting inconsistency in the association of PA with radicular pain and the need for future studies to better understand the role of PA in radicular pain. Back static muscular endurance was also inversely associated with radicular pain in the present study. Previous systematic reviews of randomized controlled trials reported that back strengthening appears to be effective in reducing pain in people with non-specific low back pain compared with usual care or no exercise but was not superior to other types of exercises such as aerobics or McKenzie exercise^21,43^. However, research specifically examining back static muscular endurance in relation to radicular pain is sparse to date. Therefore, the findings from the present study suggest that back static muscular endurance could play an important role in radicular pain and underline the need for future longitudinal studies to examine the causality of this relationship.

The main limitation of the current study is the cross-sectional design, which cannot provide evidence on whether the nature of the association found between lifestyle factors, non-specific low back pain and radicular pain is causal. Although accelerometer measurement is considered to be accurate method to assess PA^16^, the nature of this measurement is not primarily designed to identify different domains of PA, such as occupational or domestic activity and leisure time activity^44^, meaning that we were not able to classify time spent in different intensities of PA into different PA domains. In the present study, we included only those who have been examined or treated for their back pain to make sure that participants had back pain that required them to take an action, indicative of bothersome pain level. However, back pain-related disability and pain intensity, which could be greater in individuals with radiculopathy than those with non-specific low back pain, were not specifically examined in the current study. The main strength of the current study is the use of rigorous methods to assess PA with accelerometry in a large sample of people with and without low back pain at the same age.

## Conclusion

This population-based study among middle aged adults showed that non-specific low back pain and radicular pain were associated with different lifestyle and physical factors. Non-specific low back pain was associated only with the average daily number of steps, whereas abdominal obesity was the strongest determinant of radicular pain, followed by vigorous PA and back static muscular endurance. The findings from this study contribute to better understand the role of lifestyle factors in non-specific low back pain and radicular pain. Future longitudinal studies are required to explore whether these associations are causal.

## Data Availability

The data that support the findings of the present study are available from the Northern Finland Birth Cohort (NFBC) center^29^. Permission to use the data can be applied for research purposes via electronic material request portal. In the use of data, we follow the EU general data protection regulation (679/2016) and Finnish Data Protection Act. The use of personal data is based on cohort participant’s written informed consent at his/her latest follow-up study, which may cause limitations to its use. Please, contact NFBC project center (NFBCprojectcenter(at)oulu.fi) and visit the cohort website for more information.
